# Age-related immune-modulating properties of seminal fluid that control the severity of asthma are gender specific

**DOI:** 10.18632/aging.101773

**Published:** 2019-01-24

**Authors:** Yuichi Niikura, Takashi Ishii, Jurika Murakami, Tomoya Narita, Yoko Fujita, Hiroaki Negishi, Yuji Taketani, Naomi Yamashita

**Affiliations:** 1 Department of Pharmacotherapy, Research Institute of Pharmaceutical Sciences, Musashino University, Tokyo 202-8585, Japan; 2 Women’s Clinic Oizumi Gakuen, Tokyo 178-0063, Japan

**Keywords:** aging, asthma, gender difference, seminal fluid

## Abstract

Reproductive organs play a pivotal role in asthma development and progression, especially in women. Endocrine environment changes associated with the menstrual cycle, pregnancy, and menopause can exacerbate the clinical features of asthma. Factors secreted by reproductive organs may be responsible for the gender difference and age-related changes in adult asthma. Here, we show that mammalian seminal fluid has anti-asthma effects exclusively in females. Exposure to murine seminal fluid markedly reduced eosinophilic airway inflammation in 2-month-old female mice upon ovalbumin inhalation. The anti-asthma effect with seminal fluid from 10-month-old males was double that with fluid from 2-month-old males, suggesting that it depended on male sexual maturation. We further found that seminal fluid from middle-aged human volunteers had beneficial effects in asthmatic female mice; these effects were associated with transcriptional repression of osteopontin and IL-17A, which are poor prognostic factors for asthma. In 2-month-old male mice, however, human seminal fluid failed to decrease asthmatic features and even enhanced osteopontin and IL-17A transcription. Our data demonstrate that age-related seminal fluid exerts opposing effects in asthmatic male and female mice. These findings may help the development of novel approaches to control the prevalence and age-related progression of asthma in women.

## Introduction

There is compelling evidence from epidemiological studies that the incidence of asthma is greater in women than in men, and that women are more likely than men to develop severe asthma, characterized by persistent airway inflammation with poor response to steroid therapy [1,2]. This gender bias suggests that the reproductive system plays an active role in the pathogenesis of asthma. In fact, 30%–40% of females with asthma experience exacerbation of their asthmatic symptoms during the premenstrual period or pregnancy [3–6]. Poorer response to corticosteroid-based therapy in women of reproductive-age and worsening of asthma in post-menopausal women receiving hormone replacement therapy have also been reported [7,8]. Aging is recognized as a major risk factor for chronic inflammatory diseases accompanied by increasing impairment of tissue functions. Asthma in the elderly rarely goes into remission and is associated with an increased risk of exacerbation and mortality [9,10]. Given that the reproductive system is one of the earliest targets for biological aging driving systemic dysfunction, it is important to consider age-related functional changes in reproductive organs when addressing asthma control in adult patients.

Classically, recruitment of CD4^+^ T helper 2 (Th2) cells has been considered integral to the development of eosinophil-mediated allergic asthma, because these cells produce pro-inflammatory cytokines, particularly interleukins 4 (IL-4), IL-5, and IL-13, which are responsible for IgE production, eosinophil infiltration, and mucus-producing cell hyperplasia, respectively [11,12]. Experimental asthma models have been used to elucidate the underlying mechanisms of the asthmatic inflammatory responses that lead to female predominance and resistance to steroid therapy [13]. The impact of reproductive organs on asthma pathogenesis has also been studied in gonadectomized animals. Accumulating evidence in the ovalbumin (OVA)–induced asthma model has revealed that levels of Th2-related cytokines are higher in asthmatic adult female mice than in their male counterparts; this elevation causes increased eosinophilic airway inflammation and mucus-producing-cell hyperplasia [14–22]. Importantly, the accelerated asthmatic immune response in female mice is prevented by ovariectomy owing to a decline in antigen sensitization [14,16,18]. In contrast, castration of adult male mice increases asthmatic features to levels equivalent to those seen in adult female mice [14], suggesting that male-derived sex hormones such as testosterone have an anti-inflammatory/estrogen function. In support of this concept, several studies have demonstrated that systemic administration of testosterone significantly weakens asthma-related pathological changes in adult female mice [23,24]. Given the enhanced Th2-mediated immune response in male mice treated with estrogen [25], endocrine factors seem to contribute to the gender difference in asthma development and progression. Therefore, biological aging in reproductive organs has been suggested to influence the pathogenesis of adult asthma, but, so far, no direct evidence has been reported because of technical difficulties in generating antigen-sensitized old animals.

A distinct CD4^+^ helper T cell subset, namely Th17, is involved in the progression of asthma [22,26–28]. Th17 cells secrete the pro-inflammatory cytokine IL-17, levels of which are elevated in the sputum and bronchoalveolar lavage fluid (BALF) of asthmatic patients and are strongly correlated with disease severity [28,29]. Interestingly, IL-17 production in Th17 cells isolated from female patients with severe asthma is higher than that in Th17 cells from male patients [22]. Furthermore, ovarian hormones stimulate IL-17 production by the Th17 cells of patients with severe asthma [30]. Most remarkably, a study using an experimental model of murine asthma showed that adoptive transfer of OVA-primed Th17 cells is sufficient to induce steroid-resistant asthma in recipient animals in an IL-17-dependent manner [22]. Because antigen-presenting dendritic cells direct T-cell differentiation by producing Th2/Th17-inducing molecules (e.g., OX40L for Th2; IL-6, TGF-β and osteopontin for Th17), functional alteration of dendritic cells is suspected as a cause of insensitivity to steroid-based therapy as well as female predominance of asthma [31–33].

Seminal fluid is produced by male reproductive organs, including the testes, epididymis, seminal vesicles, and prostate. Secretions of these organs, which are biochemically distinct for each organ, support the complex events that occur in the uterus, such as fertilization and implantation. Fructose and citric acid support sperm mobility and survival. Prostaglandins, complement inhibitors, TGF-β, and defensins play central roles in local innate immune responses [34–38]. Recent studies have revealed that seminal CD38 endows dendritic cells with a tolerogenic function [36]. Tolerogenic dendritic cells secrete IL-10, which, in turn, stimulates *de novo* differentiation of immune-suppressive CD4^+^ regulatory T (Treg) cells, eventually leading to immune escape of alloantigens (i.e., sperm or a fertilized egg) for successful pregnancy [39–42]. In this study, we examined whether a “systemic” immune-modulative function of mammalian seminal fluid could control adult asthma. Specifically, we utilized OVA-sensitized young adult mice exposed to murine or human seminal fluid intraperitoneally or intravaginally and examined whether mammalian seminal fluid influenced asthmatic features upon OVA challenge in both males and females. We further asked whether mammalian seminal fluid modulates dendritic cell activation in response to OVA exposure *in vitro*. Finally, we examined a link between age-related functional changes in seminal fluid and asthma progression by using OVA-sensitized mature male mice.

## RESULTS

### Ovary-independent eosinophilic asthma development in adult female mice

Given that women who suffer from asthma are already sensitized to certain antigens, for clinical purposes it is important to determine whether the ovaries contribute to disease development and severity in antigen-sensitized animals. We therefore performed bilateral ovariectomy in young (2-month-old) adult female mice 1 week after OVA sensitization and housed them for at least 3 weeks until endogenous ovarian hormone production ceased (Figure S1A). Upon OVA inhalation, sensitized female mice with intact ovaries displayed extensive eosinophilic airway inflammation (Figure S1B, red). Although previous studies have reported that ovariectomy before antigen sensitization effectively reduces allergic responses [14,16,18], we failed to observe any change in the degree of eosinophilic airway inflammation in ovariectomized mice (Figure S1A B, purple). Histological analysis also showed strong infiltration of immune cells under the epithelial layer of the airways, as well as epithelial thickening and subepithelial fibrosis in the lungs of both intact and ovariectomized asthmatic female mice (Figure S1C, upper). Furthermore, hyperplasia of PAS-positive mucus-producing cells—a characteristic pathological alteration in asthmatic patients—was observed in the epithelial lining of the airways of both intact and ovariectomized mice (Figure S1C, lower), suggesting that ovary-derived female factors are not essential in the pathogenesis of adult female asthma once antigen sensitization has been established.

### Controlling eosinophilic female asthma with murine seminal fluid

**Figure 1:**
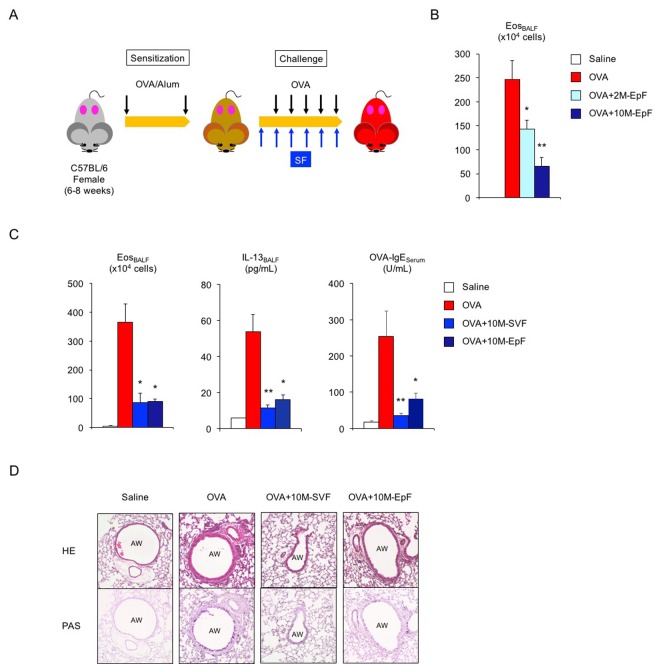
**Murine seminal fluid ameliorates asthmatic features in adult female mice.** (**A**) Schematic representation of experimental design for murine seminal fluid (SF) exposure. Young adult female mice sensitized with ovalbumin (OVA) were given murine SF intraperitoneally 30 min before OVA challenge. (**B**) Age-related functional alteration in murine SF in asthmatic female mice. Numbers of eosinophils (Eos) in bronchoalveolar lavage fluid (BALF) of asthmatic female mice exposed to epididymal fluid (EpF) from 2-month-old (2M) or 10-month-old (10M) male mice are shown. White box: control group (n = 3); colored boxes: asthma groups (n = 6–12). Data are presented as means ± SEM. **P < 0.01 and *P < 0.05 versus OVA asthma group. (**C**) Changes in Th2-cell-driven allergic responses in asthmatic female mice exposed to 10M-seminal vesicle fluid (SVF) or 10M-EpF. Eosinophil number, IL-13 section, and OVA-specific IgE antibody production are shown. White box: control group (n = 3); colored boxes: asthma groups (n = 5 each). Data are presented as means ± SEM. **P < 0.01 and *P < 0.05 versus OVA asthma group. (**D**) Representative images of airway inflammation and mucus-producing cell hyperplasia in lungs from asthmatic female mice exposed to 10M-SVF or 10M-EpF. Hematoxylin and eosin (HE, *upper*) and periodic acid-Schiff (PAS, *lower*) staining reveals immune cell infiltration and mucus-producing cell hyperplasia, respectively. AW: airway.

Next, we explored whether factors derived from male reproductive organs contribute to asthma pathogenesis and can be utilized to control female asthma (Figure 1A). Of the male accessory sex glands, we focused on the seminal vesicles and epididymis because of their major roles in semen production and maturation. Systemic exposure to murine epididymal fluid (EpF) via intraperitoneal injection successfully reduced OVA-induced eosinophilic inflammation in the lungs of 2-month-old sensitized female mice exposed to EpF from 2-month-old and 10-month-old male mice (Figure 1B, **P < 0.01 and *P < 0.05 versus OVA asthma group). Since middle-aged male seminal fluid exerted more potent anti-inflammatory activity than young adult male fluid, we used murine seminal fluid from 10-month-old male mice for further experiments. Seminal vesicle fluid (SVF) from middle-aged mice effectively suppressed eosinophilic airway inflammation in OVA-challenged asthmatic female mice (Figure 1C, left, *P < 0.05 versus OVA asthma group). Consistent with this finding, we observed a significant decrease in the levels of the Th2-related pro-inflammatory cytokine IL-13 in BALF and of OVA-specific IgE in the sera of asthmatic female mice exposed to SVF (Figure 1C, center and right, **P < 0.01 and *P < 0.05 versus OVA asthma group). Furthermore, mucus-producing cell hyperplasia and airway inflammation in asthmatic female mice were attenuated on exposure to SVF or EpF (Figure 1D). Taken together, our data indicate that murine seminal fluid from middle-aged animals suppresses antigen-induced pathological alterations in adult female mice that have been sensitized to antigen, suggesting that female asthma can be controlled by systemic exposure to seminal fluid.

### Functional conservation of human seminal fluid

**Figure 2:**
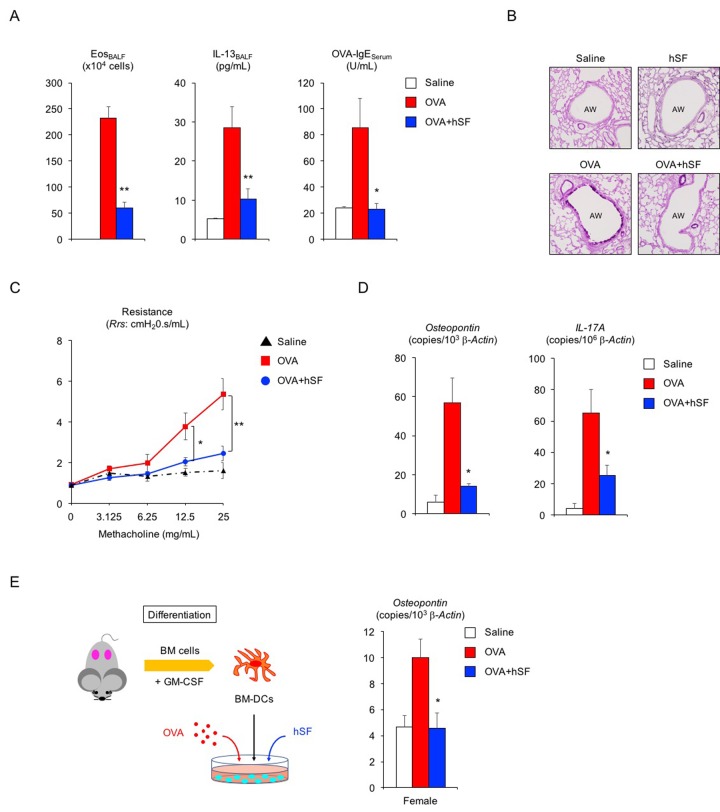
**Human seminal fluid improves pathological changes in asthmatic female mice.** (**A**) Changes in Th2-cell-driven allergic responses in asthmatic female mice exposed to human seminal fluid (hSF). White box: control group (n = 3); colored boxes: asthma groups (n = 7 each). Data are presented as means ± SEM. **P < 0.01 and *P < 0.05 versus ovalbumin (OVA) asthma group. (**B**) Representative images of PAS staining of lungs from asthmatic female mice exposed to hSF. AW: airway. (**C**) Assessment of airway hyper-responsiveness in asthmatic female mice exposed to hSF. The response to methacholine at each dose was quantified as the average of the peak measurements of airway resistance (*Rrs*). control group (n = 3, *black*); OVA asthma groups (n = 5, *red*); hSF/OVA group (n = 5, *blue*). Data are presented as means ± SEM. **P < 0.01 and *P < 0.05 versus OVA asthma group. (**D**) Transcriptional repression of *osteopontin* and *IL-17A* in lungs from asthmatic female mice exposed to hSF. White box: control group (n = 3); colored boxes: asthma groups (n = 7 each). Data are presented as means ± SEM. *P < 0.05 versus OVA asthma group. (**E**) Transcriptional repression of *osteopontin* by hSF in antigen-stimulated bone-marrow-derived dendritic cells (BM-DCs) of 2-month-old female mice. White box: control group (n = 8); colored boxes: OVA-stimulated groups (n = 8 each). Data are presented as means ± SEM. *P < 0.05 versus OVA group. BM: bone marrow, GM-CSF: granulocyte macrophage colony-stimulating factor.

To examine whether human seminal fluid (hSF) also exerts an anti-asthma effect, hSF was collected from healthy middle-aged volunteers (34 to 54 years of age). Daily exposure to hSF did not induce immune activation or histological alteration in the lungs of OVA-sensitized young adult female mice (data not shown). In addition, hSF-exposed sensitized female mice had normal reproductive cycles, as verified by vaginal smear (data not shown), indicating that hSF does not impair the ovarian function of adult female mice. hSF exposure prior to OVA challenge successfully suppressed eosinophilic airway inflammation in sensitized young adult female mice (Figure 2A, left, **P < 0.01 versus OVA asthma group). We also observed significant decreases in IL-13 secretion and OVA-specific IgE production in hSF-exposed asthmatic female mice (Figure 2A, center and right, **P < 0.01 and *P < 0.05 versus OVA asthma group). Since vaginal exposure to hSF was sufficient to improve the Th2-mediated allergic reaction (Figure S2), insemination through sexual intercourse may provide a systemic benefit to adult females with asthma.

Declines in pulmonary function in patients with asthma are mostly due to structural alterations in the lung tissue. Therefore, we next examined whether hSF could prevent pathological and functional alterations in the lungs. Histological analysis showed that hSF exposure reduced hyperplasia of PAS-positive mucus-producing cells in the airways of asthmatic female mice (Figure 2B). Furthermore, airway hyper-responsiveness to methacholine—a hallmark clinical manifestation of asthma—was significantly decreased by hSF exposure in asthmatic female mice (Figure 2C, **P < 0.01 and *P < 0.05 versus OVA asthma group), indicating that mammalian seminal fluid restores the lung compliance of asthmatic females.

Osteopontin is a pro-inflammatory cytokine involved in asthma pathogenesis, and its level in the serum or sputum of asthma patients is positively correlated with disease severity [43–46]. Accordingly, we tested whether hSF influences osteopontin expression in asthmatic adult female mice. Real-time PCR revealed a significant increase in *osteopontin* mRNA transcripts in the lungs of asthmatic female mice, but this increase was completely repressed by hSF exposure prior to OVA challenge (Figure 2D, left, *P < 0.05 versus OVA asthma group). Similarly, we found that hSF repressed the transcriptional induction of IL-17A in the lungs of asthmatic female mice (Figure 2D, right, *P < 0.05 versus OVA asthma group). Given the pivotal role of dendritic-cell-derived osteopontin in Th17 differentiation [32,33], together with the transcriptional repression of both the *osteopontin* and *IL-17A* genes by hSF, we surmised that hSF decreases *osteopontin* gene expression in antigen-stimulated dendritic cells. Bone marrow cells collected from young adult female mice were cultured in the presence of GM-CSF to obtain bone-marrow-derived dendritic cells (BM-DCs); this was followed by OVA stimulation with or without hSF. Transcriptional induction of the *osteopontin* gene in OVA-stimulated BM-DCs was completely repressed in the presence of hSF (Figure 2E, *P < 0.05 versus OVA group). Collectively, these results suggest that mammalian seminal fluid ameliorates the antigen-stimulated immunological reaction, at least in part, through the suppression of dendritic cell activity.

### Possible negative impact of seminal fluid in asthmatic male mice

**Figure 3:**
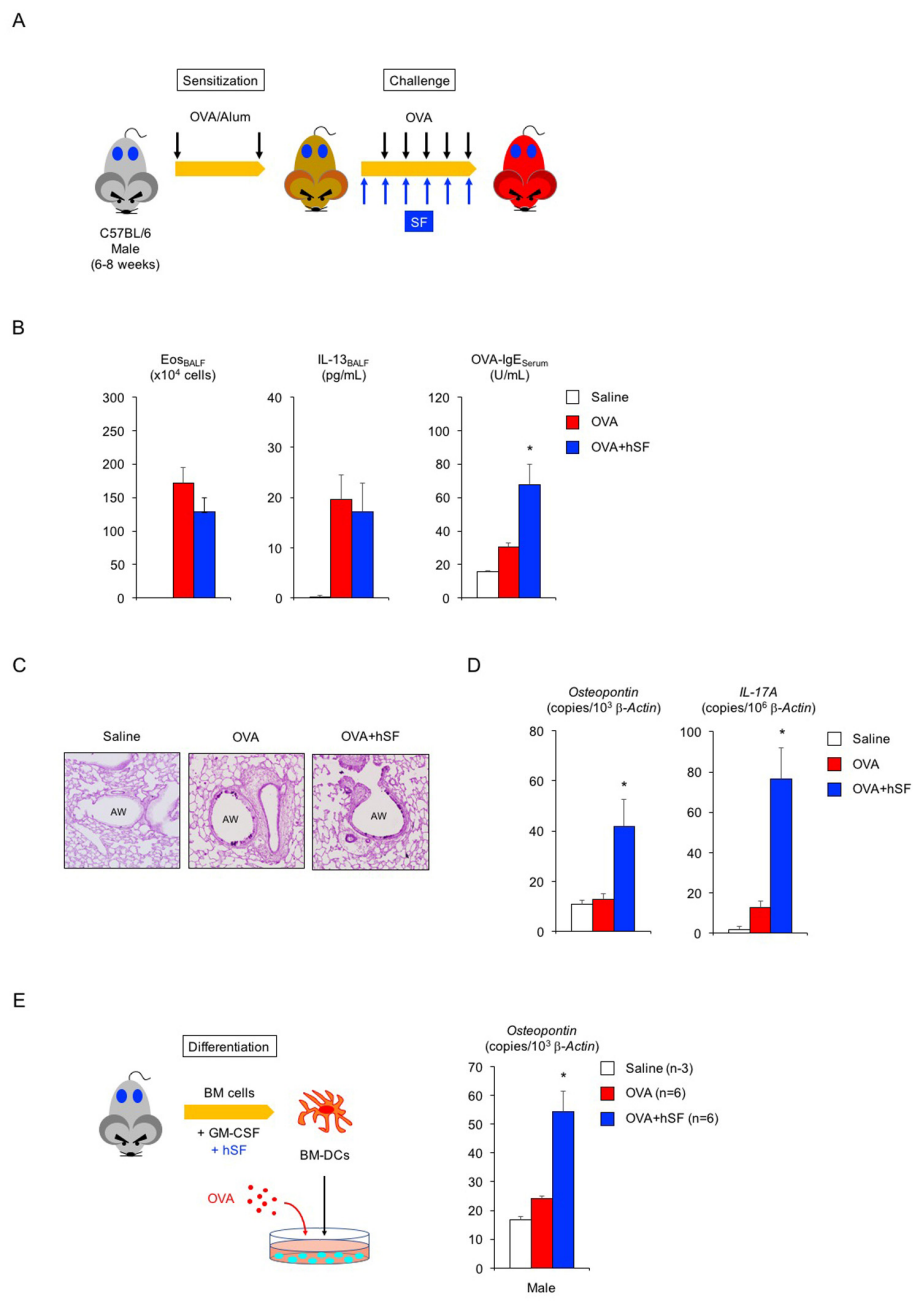
**Human seminal fluid does not improve pathological changes in asthmatic male mice.** (**A**) Schematic representation of experimental design for human seminal fluid (hSF) exposure. Young adult male mice sensitized with ovalbumin (OVA) were given hSF intraperitoneally 30 min before OVA challenge. (**B**) Changes in Th2-cell-driven allergic responses in asthmatic male mice exposed to hSF. White box: control group (n = 3); colored boxes: asthma groups (n = 5–7). Data are presented as means ± SEM. *P < 0.05 versus OVA asthma group. (**C**) Representative images of PAS staining of lungs of asthmatic male mice exposed to hSF. AW: airway. (**D**) Transcriptional induction of *osteopontin* and *IL-17A* in lungs of asthmatic male mice exposed to hSF. White box: control group (n = 3); colored boxes: asthma groups (n = 5 - 7). Data are presented as means ± SEM. *P < 0.05 versus OVA asthma group. (**E**) Transcriptional induction of *osteopontin* by hSF in antigen-stimulated BM-DCs of 2-month-old male mice. White box: control group (n = 3); colored boxes: OVA-stimulated groups (n = 6 each). Data are presented as means ± SEM. *P < 0.05 versus OVA group.

**Figure 4:**
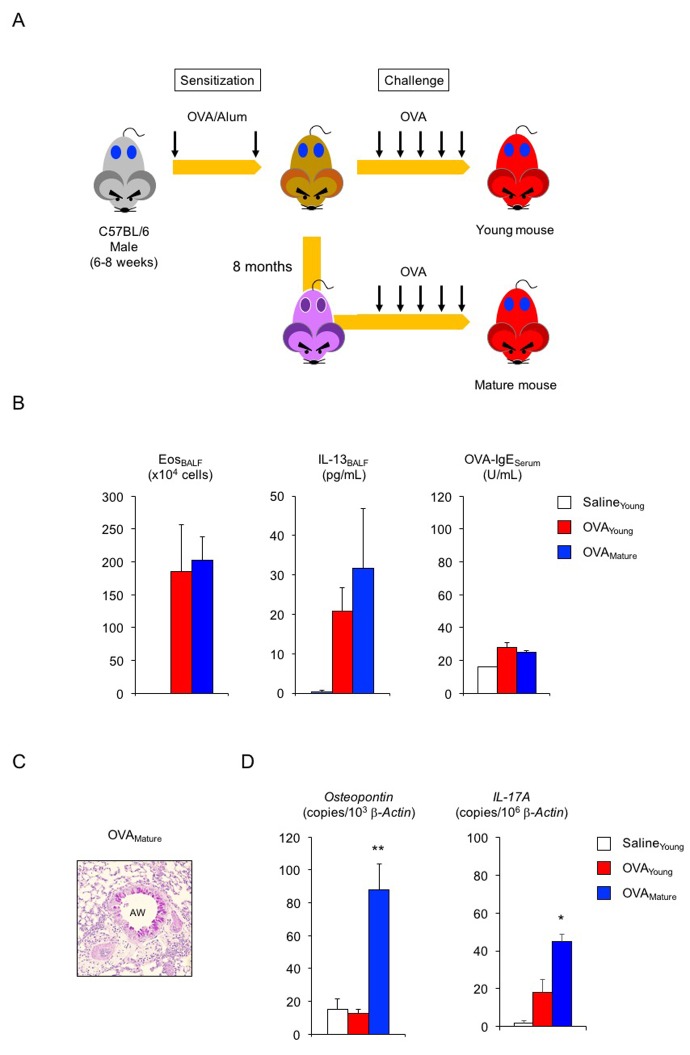
**Enhanced transcription of *osteopontin* and *IL-17A* in lungs of asthmatic mature male mice.** (**A**) Schematic representation of experimental design for asthma induction in mature male mice. Sensitized young adult male mice were challenged with ovalbumin (OVA) at 10 months of age. (**B**) Changes in Th2-cell-driven allergic responses in asthmatic mature male mice. White box: control group (n = 3); colored boxes: asthma groups (n = 5 - 7). (**C**) Representative images of PAS staining of lungs from asthmatic mature male mice. AW: airway. (**D**) Enhanced transcription of *osteopontin* and *IL-17A* in lungs of asthmatic mature male mice. White box: control group (n = 3); colored boxes: asthma groups (n = 5–7). Data are presented as means ± SEM. **P < 0.01 and *P < 0.05 versus young OVA asthma group.

Lastly, we asked whether hSF would exert a similar beneficial function in asthmatic male mice (Figure 3A). In the absence of exogenous seminal fluid, the allergic immune reaction of young (2-month-old) adult male mice was lower than that of young adult females (Figure 3B, compared with Figure 2A): 22% reduction in eosinophilic airway inflammation (*P* < 0.1 versus asthmatic female mice), 45% reduction in IL-13 secretion (*P* < 0.1 versus asthmatic female mice), and 75% reduction in OVA-IgE production (*P* < 0.01 versus asthmatic female mice). Histological analysis showed sporadic staining of PAS-positive mucus-producing cells in the airways of asthmatic male mice (Figure 3C). However, all asthmatic features tested, except for OVA-IgE production, remained unchanged upon hSF exposure. Consistent with these findings, we found reduced transcription of the *osteopontin* and *IL-17A* genes in asthmatic male mice compared with asthmatic female mice in the absence of exogenous seminal fluid (Figure 3D, red, compared with Figure 2D, red). Unexpectedly, however, we observed enhance transcriptional induction of these molecules in the lungs of asthmatic male mice exposed to hSF (Figure 3D, blue). This observation was reproduced in our *in vitro* assay system, in which OVA-induced *osteopontin* transcription was enhanced in BM-DCs derived from 2-month-old male mice and exposed to hSF throughout their differentiation process to mimic the endocrine environment of mature males *in vitro* (Figure 3E). These data strongly support the concept that age-related changes in seminal fluid shift the immune environment in males from anti-inflammatory to pro-inflammatory. To address this point, we produced asthmatic mature male mice by sensitizing 2-month-old mice with OVA and performing an OVA challenge when they reached 10 months of age (Figure 4A). Consistent with the data shown in Figure 3D, we observed a significant increase in the antigen-induced transcription of *osteopontin* and *IL-17A* in the lungs of asthmatic mature male mice (Figure 4D), but no signs of exacerbation of the asthmatic response were detected (Figure 4B, 4C).

## DISCUSSION

Beneficial functions of male-derived factors in mammalian females have been reported. Heterosexual parabiotic female mice—female mice that are joined to male mice and thereby continuously exposed to male-derived factors through shared blood circulation—exhibit enhanced *de novo* oocyte production in their ovaries [47]. Also, several studies have demonstrated that seminal fluid induces the production of immune-suppressive dendritic cells by the immune system of adult female mice in association with Treg proliferation in the uterus, giving rise to a tolerogenic local environment for successful pregnancy through the protection of the sperm and fertilized egg [40,41]. In the current study, we discovered a systemic physiological role for seminal fluid in female mammals. Artificial insemination of human seminal fluid via intraperitoneal or intravaginal injection attenuated allergic immune reactions and thereby improved asthmatic features in 2-month-old (young adult) female mice (Figures 1, 2, and Figure S2). Because angiogenesis in the female reproductive tract takes place at ovulation in an estrogen-dependent manner, absorption of seminal fluid factor(s) is expected to be maximal in the state of estrus. Therefore, insemination via sexual intercourse—a system we have adopted through the process of evolution—may be designed to provide physiological benefits to women. This idea could open new frontiers in sexual health medicine, as well as in women’s health medicine. However, seminal fluid injection failed to decrease the Th2-driven allergic reaction in 2-month-old male mice because of the lower basal immune response against antigens compared with that of female mice; this lower response may mask any potential beneficial function of the exogenous seminal fluid (Figure 3). Given that the seminal fluid of 2-month-old male mice weakened airway inflammation in asthmatic female mice (Figure 1), young-adult-male-derived factor(s) may act as circulating endogenous immune modulator(s) in mammalian males. In support of this concept, castration of young adult male mice exacerbated the antigen-induced Th2 immune reaction to a level equivalent to that experienced by asthmatic young adult female mice [14].

**Figure 5:**
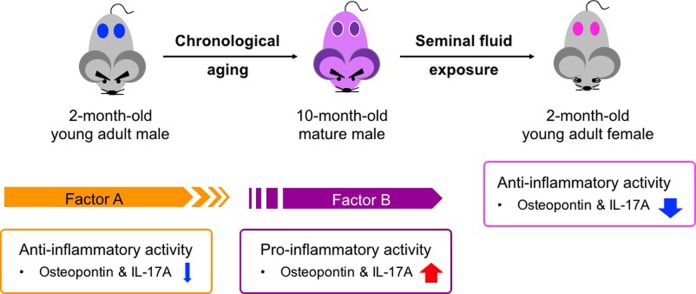
**Age-related functional alteration of seminal fluid in pathogenesis of adult asthma.** Factor A in young adult males exerts anti-inflammatory activity and may contribute to a low basal immune response compared with that in young adult females (Figure 1C and Figure 3B–D). In contrast, Factor B presents in sexually mature males and seems to exert pro-inflammatory activity (Figure 3D and Figure 4D). Both Factors A and B exhibit anti-inflammatory functions in young adult asthmatic female mice (Figure 1C). The presence of these two factors is likely associated with the gender bias and age-related progression of adult asthma.

On the basis of our findings of age-dependent functional changes in seminal fluid, we speculate that there are two types of male-derived factors (Figure 5): Factor A, a young-male-derived factor, and Factor B, a mature-male-derived factor. Factor A exerts anti-inflammatory effects that result in reduced asthmatic features in males. Since several studies have demonstrated the anti-inflammatory activity of testosterone (a major steroid hormone present in seminal fluid), testosterone is a strong candidate for Factor A. In fact, seminal fluid is enriched with free testosterone, at concentrations approximately 2 and 130 times those in the plasma of men and women, respectively [approximately 690 pg/mL in seminal fluid (67% in free form), 4870 pg/mL in male plasma (4.8% in free form), and 288 pg/mL in female plasma (1.2% in free form)] [48,49]. Taking into consideration a dilution effect on the seminal fluid components by the circulating blood after absorption (in this study, systemic administration of 200 μL of a 50-fold dilution of human seminal fluid into no less than 1 mL of circulating blood would result in at least a 250-fold dilution of the original fluid), seminal fluid exposure may not be sufficient to increase the plasma concentration of free testosterone in women to an equivalent level to that in men, suggesting the presence of other compounds highly enriched in semen, other male-specific factors, or both. In contrast, Factor B, which is derived from mature males, exhibits pro-inflammatory effects in 2-month-old male mice but exerts strong anti-asthma effects in their female counterparts. In the present study, we showed that human seminal fluid from middle-aged volunteers strongly repressed antigen-induced transcription of *osteopontin* and *IL-17A* in young adult female mice, in association with a reduction in asthmatic features (Figure 2). In young adult male mice, however, transcription of these molecules was enhanced by exogenous mature male seminal fluid (Figure 3). Interestingly, age-dependent functional alteration of male-derived factor(s) has been reported in parabiotic female mice joined to either 2-month-old or 24-month-old male mice. Circulating factors in both young adult and mature male mice accelerate postnatal oogenesis in the ovaries of their female partners, but factor(s) from young adult male mice fail to maintain newly formed oocytes owing to accelerated oocyte clearance (follicular atresia) [47]. The underlying mechanisms by which the age-related alteration in seminal fluid influences the physiological and pathological functions of mammalian females remains to be addressed; nonetheless, our study identified the dendritic cell as one of the seminal-fluid target immune cells that respond to antigens differently in males and females (Figure 2 and Figure 3). Given the critical roles of dendritic cells in the age-associated progressive decline in immune responses and the development of female-predominant autoimmunity, we believe that mammalian seminal fluid may govern aging and gender-predominant disease development throughout adult life.

The gender differences in immune responses are linked to the difference in the sex chromosomes of males and females [50]. Many of the genes on the X chromosome regulate immune cell function autonomously and play important roles in the development of immune disorders such as systemic lupus erythematosus [51]. These genes include asthma-related molecules ranging from pattern recognition receptors (*e.g.,* Tlr7 [52–55]) to transcription factors (*e.g.,* Foxp3 [56]). Females carry both paternal and maternal X chromosomes with different single nucleotide polymorphisms. Furthermore, females are mosaics of cells expressing either paternal or maternal X-linked genes, giving rising to a greater diversity to defend the body against infectious substances than is the case with the uniform cell populations of males carrying the maternal X chromosome [50]. Although such genetic differences between the sexes may explain the gender bias in the prevalence and severity of adult asthma, the impact of individual genetic background was minimized in our study by using an inbred mouse strain, thereby emphasizing the major contribution of extrinsic factors controlled by reproductive organs to sex differences in the pathogenesis of asthma.

Female-derived factors contribute to the development and progression of inflammatory diseases. We previously demonstrated the female-specific augmentation of experimental emphysema in 2-month-old mice [57]. We found that ovariectomy significantly reduced acute neutrophilic airway inflammation and subsequent alveolar destruction in elastase-treated female mice, indicating that the ovaries actively contribute to the pathogenesis of emphysema in adult females. Similarly, other murine experimental asthma studies have reported a significant decrease in the features of clinical asthma in ovariectomized adult female mice [14,16,18]. Therefore, it seems that the ovary is a promising therapeutic target for these inflammatory diseases in women. Nonetheless, this approach may not be a realistic clinical option because of the increased risk of impaired quality of life due to dysfunction of the female endocrine and reproductive systems. Our study explicitly explored a new trait of seminal fluid as it relates to the female predominance of asthma, and asthma control, without compromising ovarian function. Our future studies will focus on identifying the seminal fluid factors and their target molecules that alter dendritic cell function, and on searching for seminal fluid target cells. We believe that these studies will unveil hidden beneficial functions of seminal fluid in women.

## MATERIALS AND METHODS

### Animals

Wild-type C57BL/6Ncr mice (6 weeks of age) were purchased from the Sankyo Lab Service Corporation, Inc. (Tokyo, Japan). Mice were housed in a specific pathogen-free environment in groups of 4 or 5 per cage and allowed access to food and water ad libitum. Animal care and experimental procedures were approved by the Institutional Animal Care and Use Committee of Musashino University (H25-13011, H26-14011, H27-15011, H28-16005, H29-17007, and 05-A-2018 for animal ethic number) and were performed in accordance with *Guide for the Care and Use of Laboratory Animals*, 8^th^ edition, NIH (https://grants.nih.gov/grants/olaw/guide-for-the-care-and-use-of-laboratory-animals.pdf).

### Antigen sensitization and challenge

All animals were sensitized intraperitoneally with 2 μg of ovalbumin (OVA; A5503, Sigma-Aldrich, MO, USA) in conjunction with aluminum adjuvant in 0.5 mL of phosphate-buffered saline (PBS) on day 0 and day 14 [58]. One week after the second sensitization, the mice were challenged with either aerosolized saline or OVA (20 mg/mL saline) intranasally for 10 min on each of 5 days in a closed chamber by using a MicroMist nebulizer (DeVilbiss, PA, USA). In some experiments, sensitized mice were left until they reached 10 months of age. The mature sensitized mice were then challenged with OVA as described above to study the influence of sexual maturation and reproductive aging on the pathogenesis of asthma in adults.

### Ovariectomy in adult female mice

Sensitized female mice aged 8 weeks were anesthetized by intraperitoneal injection of a ketamine–medetomidine cocktail (55 mg/kg and 0.4 mg/kg, respectively) and underwent bilateral ovariectomy via a dorsolateral approach [57]. Ovariectomy was performed at least 4 weeks before the first OVA challenge to ensure the absence of endogenous ovarian hormones.

### Preparation of murine and human seminal fluid

Murine EpF and SVF were collected from wild-type male C57BL/6Ncr mice aged 10 months. Briefly, we cut the right and left epididymis into three regions each (caput, corpus, and cauda) in 2 mL of PBS. The luminal content was squeezed from the epididymal tubules by using fine forceps and then centrifuged at 10,000*g* for 5 min at room temperature to obtain sperm-free EpF. Seminal vesicle secretions were collected surgically (approximately 100 μL per male mouse), dissolved in 5 mL of PBS (final 50-fold dilution), and then centrifuged at 10,000 x*g* for 5 min at room temperature to obtain SVF. Both murine EpF and SVF were passed through a nylon membrane syringe filter (pore size = 0.45 μm) to eliminate potential contamination of debris. To obtain human seminal fluid, fresh semen samples were collected from healthy volunteers (34-year-old to 54-year-old men with normal sperm counts and no infection with pathogenic viruses/microbes, n = 7) and diluted 1:2 in 90% Isolate (Irvine Scientific, CA, USA). They were then transferred on to the top of a 90% ISolate layer and centrifuged at 200 x*g*. The upper phase obtained was immediately frozen in liquid nitrogen and stored until use. The upper phase was then diluted 1:25 in PBS (final 50-fold dilution) for intraperitoneal injection. Written informed consent was obtained from all volunteers. All experimental protocols were approved by the ethical committees of the Research Institute of Pharmaceutical Sciences, Musashino University and Women’s Clinic Ooizumigakuen (human ethics number H27-02).

### Exposure to murine and human seminal fluid

Thirty minutes before each OVA inhalation, 0.2-0.5 mL of the 50-fold-diluted murine or human seminal fluid samples was injected intraperitoneally into OVA-sensitized mice.

### Measurement of airway responsiveness to methacholine

Airway responsiveness to methacholine challenge was determined 24 h after the last OVA inhalation, as described previously [59]. Briefly, mice were anesthetized and a tracheostomy was performed to insert a metal endotracheal tube (18-gauge needle). The endotracheal tube was connected to a computer-controlled volume ventilator (Flexi vent, SCIREQ, QC, Canada) to measure lung function. After the assessment of baseline measures of airway resistance in response to nebulized saline, changes in these parameters in response to challenge with increasing concentrations of nebulized methacholine (3.125, 6.25, 12.5, and 25 mg/mL) were analyzed.

### Preparation of peripheral blood and BALF

Twenty-four hours after OVA challenge, mice were anesthetized as described above, and whole blood was obtained by heart puncture using a heparinized syringe to determine serum OVA-specific IgE levels. BALFs were obtained by cannulating the trachea through a small incision and flushing with 2 mL of saline; the fluids were then centrifuged at 540 x*g* for 10 min at 4 °C. The supernatant was collected for quantification of IL-13, and the pelleted bronchoalveolar lavage cells were resuspended in 1 mL of PBS to determine the total cell number by using a hemocytometer. To obtain counts of specific inflammatory cell types, no more than 50,000 cells from each BAL fluid were spun at 640 rpm for 2 min at room temperature onto glass microscope slides by using a Shandon Cytospin 4 (Thermo Electron, MA, USA). The cells were then stained with Diff-Quik (International Reagents Corporation, Osaka, Japan). At least 200 cells per mouse sample were counted; the total number of eosinophils was obtained by multiplying the percentage of eosinophils by the total number of cells.

### Histology

Left lung lobes were collected after BALF preparation, fixed in 4% (w/v) buffered paraformaldehyde phosphate (Wako Pure Chemical Industries, Ltd., Osaka, Japan) for 24 h at 4 °C, and embedded in paraffin blocks. Coronal sections (8 μm thick) were prepared and stained with hematoxylin and eosin to examine inflammatory cell infiltration of the airway epithelium or PAS to examine mucus-producing cell hyperplasia.

### Measurement of serum OVA-specific IgE and BALF IL-13

Serum OVA-specific IgE antibody levels were measured by using ELISA as described previously [58]. Briefly, OVA-coated 96-well plates (Maxisorp, Nunc, Roskilde, Denmark) were prepared, and then serially diluted serum samples (1:10–1:640) were added. The bound OVA-specific IgE antibody was labeled with a biotinylated rat anti-mouse IgE antibody (Yamasa Shoyu Co. Ltd., Chiba, Japan) and then treated with horseradish peroxidase-avidin (Dako Co., CA, USA). The chromogenic substrate 3,3’,5,5’-tetramethylbenzidine (BioFix, MD, USA) was added to the plate and the absorbance was measured with a Microwell plate reader (Tecan Group Ltd., Männedord, Switzerland) at a wavelength of 450 nm. The antibody titers of the samples were normalized against pooled standards generated in our laboratory and were expressed as units per milliliter (U/mL). The concentration of IL-13 in the BALF was determined by using a commercially available ELISA kit (R&D Systems, MN, USA) according to the manufacturer’s instructions.

### Preparation of bone marrow-derived dendritic cells and antigen stimulation

Bone marrow cells were collected from femurs and tibias of 2-month-old male or female mice and cultured for 5–7 days in Petri dishes in RPMI1640 medium supplemented with 10% fetal calf serum, antibiotics, and 10 ng/mL GM-CSF to obtain BM-DCs. The BM-DCs were then stimulated with full-length OVA (500 μγ/mL) for 24 h to analyze the transcription level of the *osteopontin* gene. The effects of hSF on *osteopontin e*xpression in BM-DCs were examined by pre-incubating the cells with 10 μL of 10-fold-diluted hSF for 30 min before OVA stimulation. Total RNA was purified form BM-DCs by using a NucleoSpin kit (Takara, Tokyo, Japan) according to the manufacturer’s instructions.

### Real-time PCR

Right lung lobes were frozen in liquid nitrogen immediately after isolation and were then fractured with a Multi Bead Shocker (Yasui Kikai Co., Osaka, Japan). Total RNA was extracted from the lung powder as described previously [57]. For cDNA synthesis, 1.5 μg of total RNA was reverse-transcribed by using PrimeScript (Takara). Real-time PCR was performed for the genes encoding Foxp3, IL-17a, and osteopontin by using SYBR Premix Ex Taq II (Takara) in a total volume of 20 μL; β-actin served as the internal control. To calculate absolute copy numbers of target genes, were used serially diluted plasmids containing the target gene cDNAs to generate a standard curve for each target gene. Melting temperature was used to confirm the specificity of the reactions. The following forward and reverse primers were used:

*β-actin* (forward, 5’-CTC CTA GCA CCA TGA AGA TCA-; reverse, 5’-CCT GCT TGC TGA TCC ACA TC-3’)

*IL-17A* (forward, 5’- CGT CAC CCT GGA CTC TCC A-3’; reverse, 5’- CAG AAT TCA TGT GGT GGT CCA-3’)

*Osteopontin* (forward, 5’-AGA ATC TCC TTG CGC CAC AG-3’; reverse, 5’-ATC GTC ATC ATC GTC GTC CAT-3’)

### Statistics

Data from experimental replicates were pooled and are presented as means ± SEM. All data were statistically analyzed by using Kruskal-Wallis tests with Dunn’s post-tests. A value of *P* < 0.05 was considered significant.

## Supplementary Material

Supplementary Figures

Supplementary Methods
