# Supplementary material for: Age-related immune-modulating properties of seminal fluid that control the severity of asthma are gender specific
Source: Aging (Albany NY). 2019 Jan 24;11(2):707–23. doi: 10.18632/aging.101773 (PMC6366957; doi:10.18632/aging.101773)
Supplement: Supplementary Figures [file aging-11-101773-s001.pdf]

SUPPLEMENTARY FIGURES

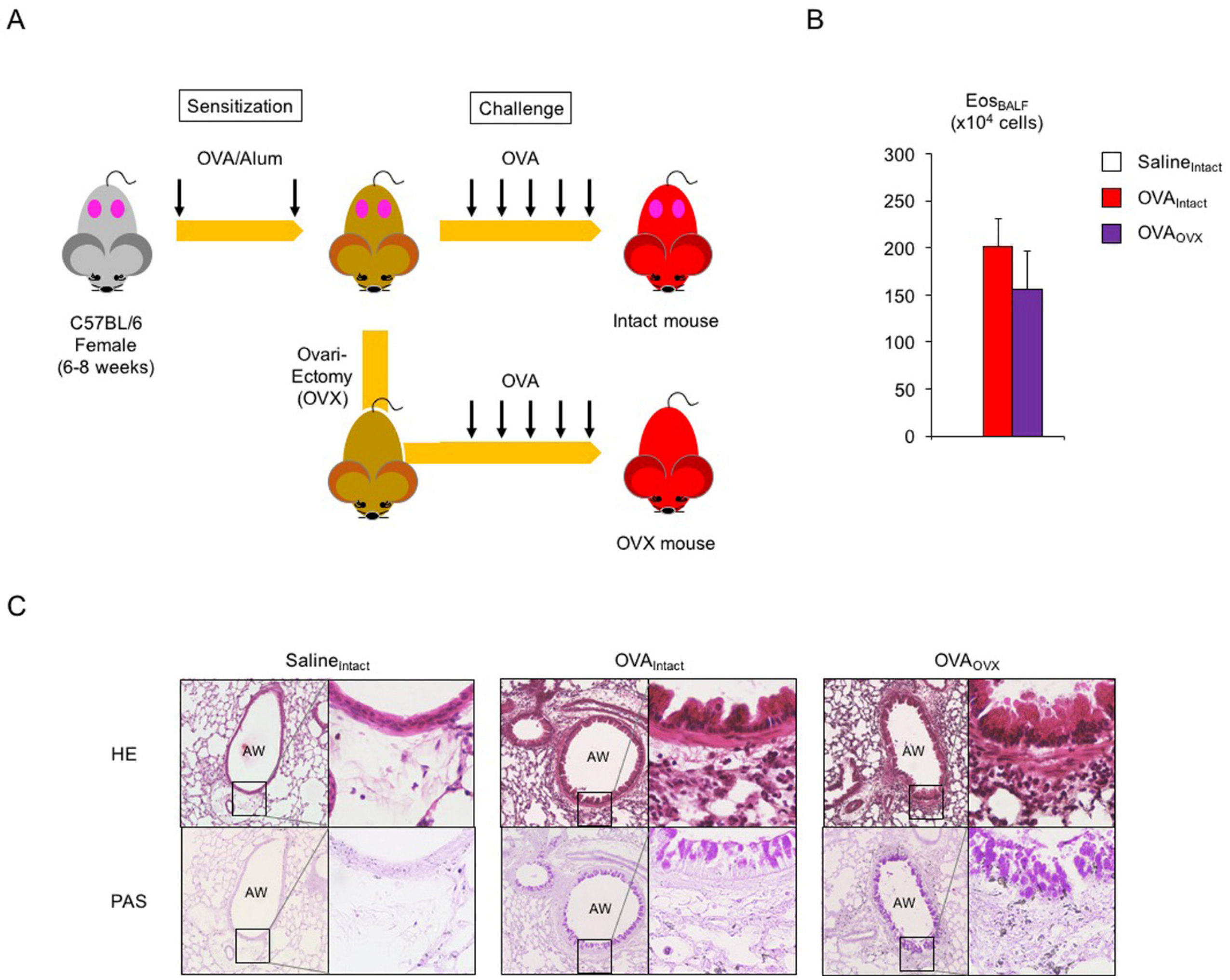

Supplementary Figure 1. Ovariectomy in sensitized adult female mice does not improve asthma development upon antigen inhalation. (**A**) Schematic representation of the experimental design for ovariectomy (OVX). Bilateral OVX was performed in 2-month-old young adult female mice sensitized with ovalbumine (OVA). Sensitized OVX mice were then challenged with OVA 3 weeks after the surgery. (**B**) Numbers of eosinophils in bronchoalveolar lavage fluid (BALF) of asthmatic OVX mice. White boxs: Saline groups (n = 3 each); Colored boxs: asthma groups (n = 6 - 8). Data are presented as means ± SEM. (**C**) Representative images of lungs from asthmatic OVX mice. Haematoxylin-Eosin (HE, *upper*) and Periodic acid-Schiff (PAS, *lower*) staining reveals immune cell infiltration and mucus-producing cell hyperplasia, respectively. Magnified images are shown on the right of each panel. AW: airway.

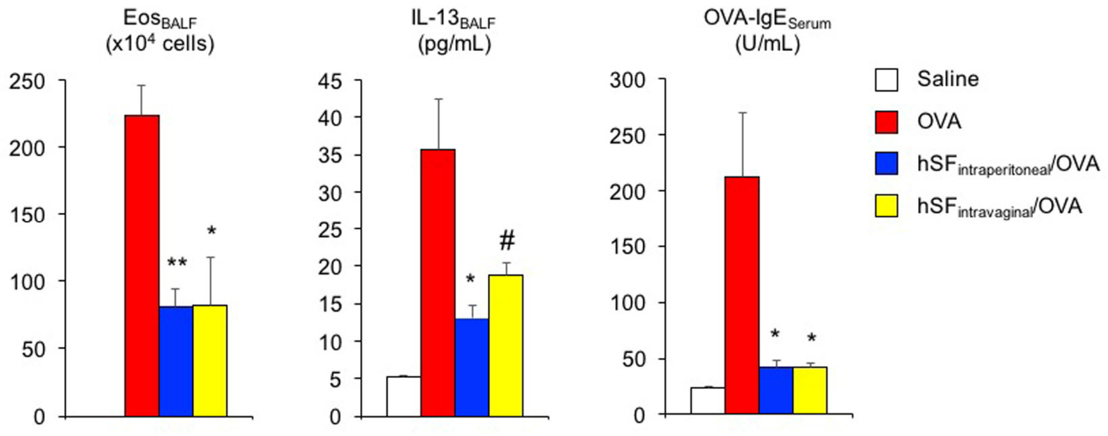

Supplementary Figure 2. Improvement of asthmatic features in asthmatic female mice exposed to human seminal fluid intravaginally. Changes in Th2 cell-driven allergic responses in asthmatic female mice exposed to human seminal fluid (hSF). White boxs: control group (n = 3); Colored boxs: asthma groups (n = 5 - 10). Data are presented as means ± SEM. **$P < 0.01$, *$P < 0.05$, and #$P < 0.1$ versus OVA asthma group.
