# Supplementary material for: Age-related immune-modulating properties of seminal fluid that control the severity of asthma are gender specific
Source: Aging (Albany NY). 2019 Jan 24;11(2):707–23. doi: 10.18632/aging.101773 (PMC6366957; doi:10.18632/aging.101773)
Supplement: Supplementary Methods [file aging-11-101773-s002.pdf]

SUPPLEMENTARY METHODS

Intravasinal exposure to murine and human seminal fluid

Fresh semen samples collected from healthy volunteers (34-year-old to 54-year-old men with normal sperm count and no infection to pathogenic viruses/microbes, n = 7) were diluted 1:2 in 90% Isolate (Irvine Scientific, CA, USA) and then transferred on the top of 90% Isolate layer, followed by centrifugation at 200 x*g*. The upper phase obtained was immediately frozen in liquid nitrogen and stored until use. For intravaginal injection, the upper phase was diluted 1:2.5 in PBS (final 5-fold dilution) and sterile cotton tampons carrying 20 μL of the 5-fold diluted human seminal fluid were daily placed in the vagina of OVA-sensitized female mice for 7 consecutive days, followed by additional exposure 30 min before each OVA inhalation. Mice with an Elizabeth collar were individually housed to prevent each other from grooming or chewing the cotton tampon. All experimental protocols were approved by the ethical committees at the Research Institute of Pharmaceutical Sciences, Musashino University and Women's Clinic Ooizumigakuen (human ethic number H27-02).
